# Urinary excretions of 34 dietary polyphenols and their associations with lifestyle factors in the EPIC cohort study

**DOI:** 10.1038/srep26905

**Published:** 2016-06-07

**Authors:** Raul Zamora-Ros, David Achaintre, Joseph A. Rothwell, Sabina Rinaldi, Nada Assi, Pietro Ferrari, Michael Leitzmann, Marie-Christine Boutron-Ruault, Guy Fagherazzi, Aurélie Auffret, Tilman Kühn, Verena Katzke, Heiner Boeing, Antonia Trichopoulou, Androniki Naska, Effie Vasilopoulou, Domenico Palli, Sara Grioni, Amalia Mattiello, Rosario Tumino, Fulvio Ricceri, Nadia Slimani, Isabelle Romieu, Augustin Scalbert

**Affiliations:** 1 Nutrition and Metabolism Unit, International Agency for Research on Cancer (IARC), Lyon, France; 2 Unit of Nutrition and Cancer, Epidemiology Research Program, Catalan Institute of Oncology, Bellvitge Biomedical Research Institute (IDIBELL), Barcelona, Spain; 3 Inserm, Centre for Research in Epidemiology and Population Health, and the Université Paris-Sud, and the Institute Gustave Roussy, Villejuif, France; 4 Division of Cancer Epidemiology, German Cancer Research Center (DKFZ), Heidelberg, Germany; 5 Department of Epidemiology, German Institute of Human Nutrition Potsdam-Rehbrücke, Nuthetal, Germany; 6 Hellenic Health Foundation, Athens, Greece; 7 WHO Collaborating Center for Nutrition and Health, Unit of Nutritional Epidemiology and Nutrition in Public Health, Dept. of Hygiene, Epidemiology and Medical Statistics, University of Athens Medical School, Greece; 8 Molecular and Nutritional Epidemiology Unit, Cancer Research and Prevention Institute – ISPO, Florence, Italy; 9 Epidemiology and Prevention Unit, Fondazione IRCCS Istituto Nazionale dei Tumori, Milan, Italy; 10 Dipartimento di Medicina Clinica e Chirurgia, Federico II University Naples, Naples, Italy; 11 Cancer Registry and Histopathology Unit, “Civic - M.P. Arezzo” Hospital, ASP Ragusa, Italy; 12 Center for Cancer Prevention (CPO-Piemonte), and Human Genetic Foundation (HuGeF), Turin, Italy

## Abstract

Urinary excretion of 34 dietary polyphenols and their variations according to diet and other lifestyle factors were measured by tandem mass spectrometry in 475 adult participants from the European Prospective Investigation into Cancer and Nutrition (EPIC) cross-sectional study. A single 24-hour urine sample was analysed for each subject from 4 European countries. The highest median levels were observed for phenolic acids such as 4-hydroxyphenylacetic acid (157 μmol/24 h), followed by 3-hydroxyphenylacetic, ferulic, vanillic and homovanillic acids (20–50 μmol/24 h). The lowest concentrations were observed for equol, apigenin and resveratrol (<0.1 μmol/24 h). Urinary polyphenols significantly varied by centre, followed by alcohol intake, sex, educational level, and energy intake. This variability is largely explained by geographical variations in the diet, as suggested by the high correlations (r > 0.5) observed between urinary polyphenols and the intake of their main food sources (e.g., resveratrol and gallic acid ethyl ester with red wine intake; caffeic, protocatechuic and ferulic acids with coffee consumption; and hesperetin and naringenin with citrus fruit intake). The large variations in urinary polyphenols observed are largely determined by food preferences. These polyphenol biomarkers should allow more accurate evaluation of the relationships between polyphenol exposure and the risk of chronic diseases in large epidemiological studies.

Polyphenols are non-nutritive plant secondary metabolites commonly found in the human diet. Over the past two decades, both experimental and epidemiological data have suggested a role of polyphenols in the prevention of chronic diseases, particularly cardiovascular diseases, type 2 diabetes and certain cancers^1^,^2^,^3^,^4^.

Dietary polyphenols constitute a large family of approximately 500 different compounds with very diverse structures and distribution in foods^5^. Daily intake of total polyphenols has been shown to vary between ~0.5 and 2 g/d across European countries^6^. Their absorption in the gut varies widely and is largely determined by their chemical structures^7^. Once absorbed, most polyphenols undergo phase II conjugation and are rapidly eliminated in urine and bile as glucuronides and sulfate esters. Non-absorbed polyphenols as well as those excreted back to the gut lumen with the bile are extensively metabolized by the microbiota, producing a range of simple phenolic compounds. Polyphenol metabolism is known to be influenced by factors such as gender, age, body mass index (BMI), renal function, gut microbiota activity, recent use of antibiotics, and genetic traits^8^,^9^. Due to these many factors that may determine polyphenol bioavailability, biomarkers may be better indicators of polyphenol exposures and better predictors of disease risk than intake measurements assessed using dietary questionnaires^3^.

To date, concentrations of polyphenols have been measured in urine or blood in a limited number of epidemiologic studies^3^,^10^. However, the range of polyphenols simultaneously measured was limited to a few compounds, most often isoflavones or lignans. Recently, we developed a new method that allows the quantification in urine of 37 polyphenols and polyphenol metabolites representative of the major polyphenol classes and subclasses^11^.

These polyphenols are measured in urine from 475 participants of the European Prospective Investigation into Cancer and Nutrition (EPIC) study. This study offers a unique opportunity to compare the urinary excretion of polyphenols in subjects from different European countries with a large variability in polyphenol intakes^6^. The influence of several lifestyle and dietary factors on urinary polyphenol concentrations is also examined.

## Material and Methods

### Study population

The EPIC study is a large cohort study with over half a million participants of both genders mostly recruited from the general population between 1992 and 2000 in 23 centres from 10 European countries^12^. Data used in the present study were derived from the EPIC calibration study (n = 36,994), in which a single 24-hour dietary recall (24-HDR) was collected from a random sample of the entire cohort^13^. In a convenience sub-sample (n = 1,386), 24-hour urine specimens were collected between 1995 and 1999^14^. Individuals who collected the 24-hour urine specimen and the 24-HDR on the same day were included for the present study (n = 475). The study was performed in accordance with the approved guidelines. Approval for the study was obtained from ethical review boards of the International Agency for Research on Cancer (IARC) and from all participating institutions. All participants provided written informed consent.

### 24-Hour urine specimen

24-Hour urine samples were collected over 2 g boric acid used as preservative and stored at −20 °C. Completeness of collection was monitored using *p*-aminobenzoic acid (PABA) given to participants in tablet form^14^.

### Urinary polyphenol measurements

Urine samples were first hydrolysed with a β-glucuronidase/sulfatase enzyme mixture and the resulting polyphenol aglycones were extracted twice with ethyl acetate. Quantitative dansylation of phenolic hydroxyl groups was carried out with either ^13^C-labelled dansyl chloride (samples) or non-labelled dansyl chloride (well-characterized reference pooled sample) as previously described^11^. Each ^13^C-dansylated sample was mixed with the ^12^C-dansylated reference sample, and the relative concentrations in samples over the reference sample were determined by UPLC-ESI-MS-MS in batches of 25 samples. Limits of quantification (LOQ) for the 37 polyphenols varied between 0.01 μM for equol and 1.1 μM for 4-hydroxyphenylacetic acid. Intra-batch coefficients of variation varied between 3.9% and 9.6% depending on polyphenols. Inter-batch variations were lower than 15% for 31 compounds and lower than 29% for 6 additional polyphenols out of the 38 tested.

### Dietary and lifestyle information

Dietary data were collected using a single 24-HDR using a harmonized methodology (EPIC-Soft)^15^. The 24-HDR was administered in a face-to-face interview. Total energy and alcohol intakes were estimated by using the standardized country-specific EPIC Nutrient Database^15^. Data on lifestyle factors, including educational level, physical activity and smoking history, were collected at baseline through questionnaires^13^,^16^. Data on age, body weight and height were self-reported by study participants during the 24-HDR interview.

### Statistical analyses

Urinary polyphenol concentrations that fell below the LOQ were set to values corresponding to half the limit of quantification^17^,^18^. Three polyphenols (procyanidins B1 and B2, and (+)-gallocatechin) were excluded from the analysis, since 98–100% of the values were <LOQ^11^. Levels of polyphenol 24-hour urinary excretion are presented as medians and 10^th^ and 90^th^ percentiles, since they had skewed distributions. Pearson correlation coefficients between excretion levels of the 34 remaining compounds were computed after log-transformation and visualized using a heatmap plot. Spearman correlation coefficients between the 34 urinary polyphenols and 110 plant-derived food groups were also calculated.

The sources of variability within the urinary polyphenol excretion pertaining to lifestyle characteristics and technical processing parameters were assessed using principal component partial R-square (PC-PR2) analysis^19^. PC-PR2 identifies and quantifies sources of variability by combining features of principal component analysis with those of multivariable linear regression analysis. In this study, the list of variables scrutinized included: age, sex, study centre, BMI (kg/m^2^), alcohol intake (g/d), educational level, smoking status, physical activity, and batch. Categorical variables were modelled using indicator variables in regression analyses. A variance threshold equal to 80% was used in the PC-PR2. Analytical missing values of urinary polyphenols were imputed using the expectation-maximization algorithm prior to PC-PR2 analysis^20^. Urinary polyphenols with a percentage of missing values greater than 20% (gallic acid and 3-hydroxyphenylacetic acid) were excluded from the PC-PR2 analysis. Kruskal-Wallis tests were used to assess differences of 34 urinary polyphenol levels according to demographic and lifestyle factors. The threshold for statistical significance was set after Bonferroni correction for the number of measured polyphenols, to a *P* value < 0.001 (0.05/34) (two-tailed).

All analyses were conducted using the R software, version R.3.1.2 (R Foundation for Statistical Computing, Vienna, Austria).

## Results

The 475 participants included in the study were 33–77 years old and mostly recruited from the general population residing in defined geographical areas in France (Paris and surroundings), Germany (Heidelberg and Potsdam), Greece (nation-wide) and Italy (Florence, Naples, Ragusa, Turin, and Varese). The percentage of women ranged from 35% (Ragusa) to 71% (Florence), except in France and Naples where only women were recruited (Table 1). Anthropometric and lifestyle characteristics are given in Table 1.

**Figure 1:**
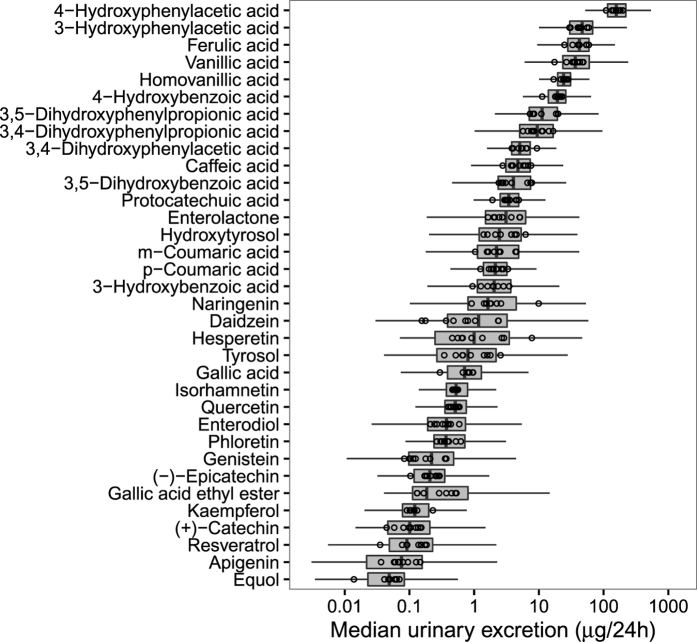
Urinary polyphenol concentrations by study centre in the EPIC cohort. Dots in the boxplot are the medians of urinary polyphenol concentrations in each centre.

Thirty four polyphenols were detected and quantified in 24-hour urine collected in the 475 subjects. Medians of urinary excretion are shown in Table 2 and Fig. 1. 4-Hydroxyphenylacetic acid was the most abundant polyphenol in urine (157 μmol/24 h), followed by 3-hydroxyphenylacetic, ferulic, vanillic and homovanillic acids, with excretion levels varying between 20 and 50 μmol/24 h. Equol, apigenin and resveratrol were found in the lowest quantities (<0.1 μmol/24 h). A high percentage of participants with urinary concentrations below the limit of quantification was observed for isorhamnetin (55%), phloretin (52%), gallic acid ethyl ester (52%), (+)-catechin (37%), (−)-epicatechin (27%), hesperetin (26%) and apigenin (25%).

**Figure 2:**
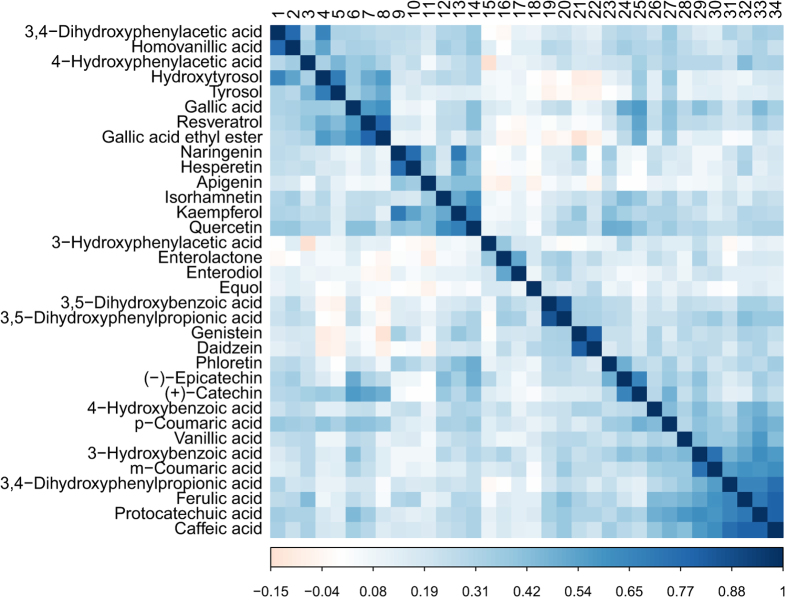
Heatmap of Pearson correlations between the log-transformed urinary polyphenol excretions in the EPIC study.

When correlations between urinary polyphenols were examined, forty-one moderate to high correlations (r > 0.5) were found (Fig. 2). The highest correlations were observed for the following compounds: 3,5-dihydroxybenzoic acid and 3,5-dihydroxyphenylpropionic acid (r = 0.86), genistein and daidzein (r = 0.83), protocatechuic acid and caffeic acid (r = 0.81), ferulic acid and caffeic acid (r = 0.80), resveratrol and gallic acid ethyl ester (r = 0.80), naringenin and hesperetin (r = 0.77), 3,4-dihydroxyphenylacetic acid and homovanillic acid (r = 0.77), and caffeic acid and 3,4-dihydroxyphenylpropionic acid (r = 0.76).

Large differences in the urinary excretion of each polyphenol were observed between subjects. PC-PR2 analysis showed that 23.5% of the total variance in urinary polyphenol excretion was explained by lifestyle and analytical factors. Study centre displayed the largest *R*_partial_^2^ value (9.6%), followed by batch (5.1%) and alcohol intake (4.1%). The remaining factors (age, sex, BMI, educational level, smoking status, and physical activity) accounted for a minor fraction of the variability (<1.2% for each factor).

Differences in polyphenol urinary excretion between study centres are illustrated in Fig. 1 and Supplemental Table 1. For example, median urinary excretion of hesperetin was 17-fold higher in Ragusa-Italy (7.8 μmol/24 h) than in France (0.46 μmol/24 h). Median excretion levels of daidzein were 15-fold higher in Heidelberg-Germany (2.38 μmol/24 h) than in Ragusa-Italy (0.16 μmol/24 h), 11-fold higher for naringenin in Ragusa-Italy (9.9 μmol/24 h) than in France (0.91 μmol/24 h), and 7-fold higher for tyrosol in Ragusa-Italy (2.56 μmol/24 h) than in Potsdam-Germany (0.35 μmol/24 h).

Variations of urinary polyphenol excretions according to other lifestyle factors were also examined. For sex, 10 urinary polyphenols were significantly more abundant in men than in women. Indeed, median urinary levels of tyrosol, hesperetin, naringenin, vanillic and 4-hydroxyphenylacetic acids were at least 1.4-fold higher in men than in women (Supplementary Table 2). For schooling level, urinary daidzein (3.1-fold change), enterolactone (1.8-fold change), gallic acid (1.6-fold change), and 4-hydroxybenzoic acid (1.2-fold change) levels were significantly lower in less educated people (none or primary school completed) compared to subjects with higher education (Supplementary Table 3). For total energy intake, higher levels of 7 polyphenols in urine (4-hydroxyphenylacetic, ferulic, vanillic, homovanillic, protocatechuic and *p*-coumaric acids, and equol) were observed in those who fell into the top tertile of energy intake (Supplementary Table 4). For BMI, only the excretion of gallic acid was significantly different across BMI subgroups (data not shown). Its level decreased with increasing BMI: 0.87 μmol/24 h for subjects with BMI <25 kg/m^2^, 0.64 μmol/24 h for subjects with BMI between 25 and 30 kg/m^2^, and 0.50 μmol/24 h for subjects with BMI ≥30 kg/m^2^. For total alcohol consumption, subjects drinking >20 g of alcohol/d showed urinary concentrations 9-fold, 7-fold, 5-fold, 4-fold, 3-fold, and 2.3-fold higher for tyrosol, gallic acid ethyl ester, resveratrol, hydroxytyrosol, (+)-catechin, and gallic acid, respectively, when compared to subjects drinking <0.1g alcohol/d (Supplementary Table 5). No significant differences were observed for the remaining factors studied: age, smoking status, and physical activity (data not shown).

Correlations between urinary excretion of specific polyphenols and intakes of 110 food groups were systema-tically studied. Plant-derived foods were considered in this analysis due to the plant origin of polyphenols. The urinary excretions of a large number of the measured polyphenols were found to be correlated to the intake of 14 of the 110 plant-derived food groups documented in the 24-HDR (Table 3)^19^,^20^. For each of these food groups, polyphenols were ranked according to their Spearman correlation coefficient. The first two to nine most highly correlated polyphenols are shown in Table 3. Correlations with 4 of these food groups need to be interpreted with caution due to the high percentage of non-consumers (>90%): olives (90.7%), berries (91.2%), grapes (96.4%), and soy products (98.1%). Correlations of polyphenols with intake of these 4 polyphenol-rich food groups were low (data not shown). In addition, correlation between urinary excretion of equol and intake of dairy products was also examined because of the known occurrence of equol in these food products^21^. Statistically significant correlations between levels of urinary equol and the intake of dairy products (r = 0.33), especially with milk (r = 0.27) and cheese (r = 0.18), were found. Correlations between intake of polyphenol-rich foods or food groups were also examined. Correlations were low (data not shown) except for olive oil and coffee intake (r = −0.48).

## Discussion

In the current study, a new analytical method was used to estimate, in an adult European population, the concentrations of 34 urinary polyphenols of all main polyphenol classes: flavonoids, phenolic acids, lignans and stilbenes. These polyphenols detected in urine after enzymatic deconjugation are either parent compounds as found in food, phenolic microbial metabolites or *O*-methylated tissular metabolites (Table 2). Far fewer polyphenols were measured in previous population studies^22^,^23^, most of them being focused on the analysis of a specific polyphenol class, such as stilbenes^24^, phytoestrogens (isoflavones and lignans)^25^, or alkylresorcinols^26^.

As expected, levels of urinary excretion varied highly between polyphenols. The most abundant urinary polyphenols detected in our study were phenolic acids formed by the microbiota: 4- and 3-hydroxyphenylacetic acids, 3,4-dihydroxyphenylacetic acid, protocatechuic acid (and their *O*-methylated metabolites: homovanillic acid and vanillic acid, respectively), 4-hydroxybenzoic acid, 3,5- and 3,4-dihydroxyphenylpropionic acids, and, 3,5-dihydroxybenzoic acid^27^, with median excretion levels ranging from 3.4 to 157 μmol/24 h. These phenolic acids are produced by microbial transformation of a wide range of dietary polyphenols^28^,^29^, as well as endogenous metabolites such as dopamine^30^ and aromatic amino acids^31^. Two hydroxycinnamic acids were also excreted in urine at high levels: caffeic acid (4.7 μmol/24 h) mainly derived from the hydrolysis of caffeoyl esters such as chlorogenic acids abundant in coffee, and ferulic acid (42 μmol/24 h) that may originate both from *O*-methylation of caffeic acid in the tissues and the hydrolysis in the gut of ferulic acid esterified to cereal cell walls^32^. Urinary levels of flavonoids, lignans, tyrosols and stilbenes were low (median excretions <3.1 μmol/24 h). These low levels are explained by either low intakes (e.g. isoflavonoids, stilbenes, lignans, tyrosols)^6^,^33^, or poor absorption (often 0.1–10% depending on the specific polyphenol)^7^. Levels of polyphenol urinary excretion were comparable to those of 11 polyphenols previously measured in a population of 53 French adults^22^.

Excretion levels of the different polyphenols showed correlations that can be explained by either co-occurrence in a given food group or by metabolic parentage. Typical examples of food co-occurrence are genistein and daidzein in soy products (r = 0.82), resveratrol and gallic acid ethyl ester in wine (r = 0.76), naringenin and hesperetin in citrus fruits (r = 0.78), tyrosol and hydroxytyrosol in olive oil (r = 0.70), (−)-epicatechin and (+)-catechin in tea, apple, wine and chocolate (r = 0.66), phloretin and quercetin (r = 0.53) and phloretin and (−)-epicatechin (r = 0.49) in apple^34^,^35^. Correlations between metabolites participating in a common metabolic pathway involve both metabolites linked through microbial catabolic reactions and *O*-methylation reactions carried out in tissues such as the liver. High correlations were observed between microbial metabolites and their precursors: 3,5-dihydroxyphenylpropionic acid and 3,5-dihydroxybenzoic acid (r = 0.86), two main metabolites of alkylresorcinols^36^, enterodiol and enterolactone (r = 0.50), *m*-coumaric acid and 3-hydroxybenzoic acid (r = 071), caffeic acid and 3,4-dihydroxyphenylpropionic acid (r = 0.74), caffeic acid and protocatechuic acid (r = 0.79), and protocatechuic acid and 3-hydroxybenzoic acid (r = 0.58). *O-*methylation reactions explain correlations between 3,4-dihydroxyphenylacetic acid and homovanillic acid (r = 0.76), quercetin and isorhamnetin (r = 0.64), protocatechuic acid and vanillic acid (r = 0.52), and caffeic acid and ferulic acid (r = 0.79). The particularly high correlation observed between caffeic and ferulic acids suggests that ferulic acid originates mainly from the *O*-methylation of caffeic acid, although the weak correlation observed with intake of non-white bread (Table 3) also supports its formation through hydrolysis of ferulic acid bound to cereal cell walls^37^.

Urinary polyphenol excretion differed widely according to study centre, with 10-fold higher changes for hesperetin, naringenin and daidzein, and 5-fold higher changes for tyrosol, resveratrol and equol. Similar magnitudes of changes in plasma concentrations between centres were observed for isoflavones (13-fold for daidzein and 8-fold for genistein) and lignans (4-fold for enterolignans) in a previous EPIC study^25^. These large variations of urinary excretions across study centres could be due to differences in dietary patterns across European countries. Polyphenols and polyphenol-rich foods are consumed diversely across centres of the EPIC study^6^, and polyphenol urinary excretion is expected to differ similarly.

In addition to study centre, polyphenol urinary excretion was found to be associated with several other sociodemographic, lifestyle and anthropometric factors, Total alcohol consumption was a relevant source of variability. Among sources of alcohol, red wine is particularly rich in polyphenols and its consumption varies widely between study centres^38^. In the current study, red wine was significantly correlated with levels of several polyphenols in urine, including gallic acid ethyl ester and resveratrol. Men also excreted more polyphenols than women, although differences were relatively small (<2.4) compared to differences by study centre or alcohol consumption. A potential explanation is that men consume more calories than women (mean 2,502 vs. 2,108 kcal/d), and higher total energy intake was shown to be positively associated with higher polyphenol intake^6^. This is consistent with the higher urinary excretion of polyphenols we observed in subjects in the highest tertile of total energy intake. Concentrations of 4-hydroxyphenylacetic, ferulic, vanillic, homovanillic and *p*-coumaric acids were higher in men and in subjects consuming more calories. Higher polyphenol excretion in men can also be explained by a higher consumption of coffee in men as compared to women (343 vs. 244 mL/d). In agreement with this interpretation, two of the compounds showing higher concentrations in men (ferulic and vanillic acids; see Table 3) were also highly correlated with coffee consumption. Education level was associated with the excretion of certain phenolic compounds. Subjects with no or only a primary level of education had lower levels of 4-hydroxybenzoic acid, enterolactone, daidzein and gallic acid than those with a higher education level. Polyphenol intake was also previously found to be higher in people with a university degree than in those without one^6^. Concentrations of gallic acid in urine were inversely associated with BMI. They were also moderately correlated with tea and wine consumption (r = 0.44 and 0.45, respectively), which are usually related to a healthier lifestyle and higher education level^39^. Dietary flavonoids, characteristic of wine and tea^34^, are also higher in subjects with lower BMI (<25 kg/m^2^) in the EPIC cohort^6^,^40^. No differences were observed by age, smoking status, and physical activity.

Correlations between urinary polyphenol excretions and food intake (Table 3) show the consistency of our analytical results and point towards the potential use of these phenolic compounds as dietary biomarkers^10^,^41^. As expected, we observed high correlations between red wine intake and the main polyphenols coming from red wine, such as gallic acid ethyl ester (r = 0.69) and resveratrol (r = 0.59)^41^,^42^. These correlations are similar to those observed with total alcohol consumption. High correlations were also observed between coffee consumption and caffeic acid (r = 0.65), and citrus fruit intake and hesperetin and naringenin (r = 0.60 and 0.56 respectively)^43^. Weaker correlations (0.31 < r < 0.45) were observed between tea intake and gallic acid, apple intake and phloretin, olive oil consumption and hydroxytyrosol and tyrosol, non-white bread intake and 3,5-dihydroxybenzoic acid and 3,5-dihydroxyphenylpropionic acid. All these phenolic compounds are known to be characteristic of the foods with which they are correlated or particularly abundant in these foods^34^ and several of them have been proposed as biomarkers of intake for these foods^41^,^44^,^45^,^46^. Correlation of urinary equol with consumption of dairy products (r = 0.33) provides new information of their dietary sources in this population. Equol, a metabolite of daidzein formed by the gut microbiota, was detected in 86% of the subjects (Table 2). Its correlation with dairy products and not soy food intake provides new evidence of its dairy origin through its formation from daidzein in the rumen of cows fed soybeans and secretion in milk^21^.

The magnitude of the correlations observed between polyphenols in urine and food intake depends on various factors, including the reliability of the dietary intake measurements, the variability of polyphenol contents in a given food, the existence of confounders such as other foods containing the same polyphenol or polyphenol precursors (see in Table 3, gallic acid correlated with both red wine and tea, ferulic acid correlated with both coffee and non-white bread, hydroxytyrosol correlated with both red wine and olive oil), and inter-individual variability in the transformation of the food parent compound to the phenolic biomarker. For these reasons, levels of correlation observed here have limited value *per se* to evaluate the usefulness of a potential biomarker. However, they are useful indicators when comparing the potential value of different biomarkers for a particular food. Polyphenols showing the highest correlations (Table 3) should also be the best predictors of food intake in this population.

This study is the first showing variations of a broad profile of urinary polyphenols in healthy European people. The present study has a number of strengths, in particular the novel analytical method based on the use of tandem mass spectrometry, which made possible the estimation of a large number of polyphenols. Another advantage was the collection of 24 h urine samples rather than spot urine samples, which is not so common in large epidemiological studies. Furthermore, methods of urine collection, sample handling and storage, and dietary assessment were highly standardized in all study centres^14^. The main limitation of the current study is that our results are not fully generalizable, since not all EPIC cohorts are population-based^12^. Another limitation is that exposure to some important polyphenols could not be measured in urine with our method (anthocyanidins and gallocatechins) or could not be measured with sufficient sensitivity (e.g. proanthocyanidin dimers not detected)^11^. Finally, no data are available regarding the effect of long term storage on the concentrations of urinary polyphenols, although a prior study has shown that urinary resveratrol concentrations remained unchanged when samples had been stored at −80 °C for 5 years^47^,^48^. However, possible degradation of test compounds in urine over time should affect similar to all participants since all samples have a long but relatively similar storage time.

In conclusion, this study shows large variations in excretions of urinary polyphenols across adult European populations, reflecting considerable variability in the consumption of polyphenol-rich foods. Some of these urinary polyphenols may also be used as dietary biomarkers for some polyphenol-rich foods, and further research in other large epidemiological studies and intervention studies is warranted for further validation. Measurement of these polyphenols in urine should allow more accurate evaluation of polyphenol exposure to reveal new associations with risk of chronic diseases in large epidemiological studies.

## Additional Information

**How to cite this article**: Zamora-Ros, R. *et al.* Urinary excretions of 34 dietary polyphenols and their associations with lifestyle factors in the EPIC cohort study. *Sci. Rep.* **6**, 26905; doi: 10.1038/srep26905 (2016).

## Supplementary Material

Supplementary Information

## Figures and Tables

**Table 1:** Centre-specific characteristics of the study population.

Centre (Country)	n	Women	Age	Never smoking	Physically inactive	University studies	BMI	Energy intake	Alcohol intake
		%	years*	%	%	%	kg/m^2^*	kcal/d*	g/d#
Ile-de-France (France)	67	100	53 (7)	67	19	43	23 (4)	2,082 (683)	9 (0–18)
Florence (Italy)	45	71	56 (6)	42	27	16	26 (4)	2,022 (546)	12 (0–21)
Varese (Italy)	51	37	57 (7)	45	8	6	25 (3)	2,525 (880)	12 (0–32)
Ragusa (Italy)	17	35	50 (7)	29	24	12	26 (4)	2,529 (999)	5 (0–25)
Turin (Italy)	42	48	53 (7)	52	36	29	25 (3)	2,439 (697)	22 (0–38)
Naples (Italy)	20	100	48 (6)	40	55	15	27 (5)	1,955 (545)	5 (0–12)
Greece	56	52	58 (11)	54	45	4	30 (4)	1,728 (659)	0 (0–8)
Heidelberg (Germany)	59	61	51 (9)	54	9	29	25 (5)	2,431 (995)	11 (1–38)
Potsdam (Germany)	118	41	54 (9)	48	31	41	27 (4)	2,212 (707)	1 (0–20)
TOTAL	475	58	54 (9)	51	26	26	26 (4)	2,200 (785)	8 (0–23)

^*^mean (standard deviation).

^#^median (25th–75th).

**Table 2:** Urinary polyphenol excretion (μmol/24 h) in 475 subjects from the EPIC cohort.

Urinary polyphenols	Polyphenol class/subclass	Origin^1^	N^2^	<LOQ (n)	Median	P10th	P90th
4-Hydroxybenzoic acid	Phenolic acids/Hydroxybenzoic acids	Microbiota	473	0	19.37	9.90	35.65
3-Hydroxybenzoic acid	Phenolic acids/Hydroxybenzoic acids	Microbiota	475	0	2.03	0.66	6.37
Protocatechuic acid	Phenolic acids/Hydroxybenzoic acids	Microbiota	475	0	3.43	1.80	6.30
Gallic acid	Phenolic acids/Hydroxybenzoic acids	Food	336	5	0.71	0.26	2.25
Vanillic acid	Phenolic acids/Hydroxybenzoic acids	Microbiota/food	464	0	36.45	14.59	93.76
3,5-Dihydroxybenzoic acid	Phenolic acids/Hydroxybenzoic acids	Microbiota	468	0	4.04	1.61	12.31
Gallic acid ethyl ester	Phenolic acids/Hydroxybenzoic acids	Food	450	234	0.19	0.08	2.65
4-Hydroxyphenylacetic acid	Phenolic acids/Hydroxyphenylacetic acids	Microbiota	474	0	156.82	91.19	324.80
3-Hydroxyphenylacetic acid	Phenolic acids/Hydroxyphenylacetic acids	Microbiota	299	11	46.72	19.93	95.50
3,4-Dihydroxyphenylacetic acid	Phenolic acids/Hydroxyphenylacetic acids	Microbiota	475	0	5.06	2.82	10.69
Homovanillic acid	Phenolic acids/Hydroxyphenylacetic acids	Microbiota/endogenous	475	0	24.08	15.63	38.70
3,4-Dihydroxyphenylpropionic acid	Phenolic acids/Hydroxyphenylpropanoic acids	Microbiota	453	0	9.45	3.24	29.02
3,5-Dihydroxyphenylpropionic acid	Phenolic acids/Hydroxyphenylpropanoic acids	Microbiota	473	0	11.19	4.60	28.44
*p*-Coumaric acid	Phenolic acids/Hydroxycinnamic acids	Food/microbiota	464	0	2.13	0.92	4.82
*m*-Coumaric acid	Phenolic acids/Hydroxycinnamic acids	Microbiota	467	8	2.22	0.55	9.57
Caffeic acid	Phenolic acids/Hydroxycinnamic acids	Food	475	0	4.75	1.98	10.60
Ferulic acid	Phenolic acids/Hydroxycinnamic acids	Endogenous/food	470	0	42.21	18.37	83.02
Kaempferol	Flavonoids/Flavonols	Food	408	35	0.12	0.05	0.30
Quercetin	Flavonoids/Flavonols	Food	444	0	0.51	0.23	1.10
Isorhamnetin	Flavonoids/Flavonols	Endogenous	462	255	0.52	0.27	1.17
Apigenin	Flavonoids/Flavones	Food	448	113	0.08	0.01	0.34
Naringenin	Flavonoids/Flavanones	Food	470	11	1.63	0.43	9.32
Hesperetin	Flavonoids/Flavanones	Food	469	122	1.00	0.16	8.29
Daidzein	Flavonoids/Isoflavonoids	Food	407	13	1.18	0.14	8.33
Genistein	Flavonoids/Isoflavonoids	Food	413	12	0.22	0.05	1.19
Equol	Flavonoids/Isoflavonoids	Microbiota	397	54	0.05	0.01	0.14
Phloretin	Flavonoids/Dihydrochalcones	Food	475	248	0.37	0.17	1.14
(+)-Catechin	Flavonoids/Flavanols	Food	452	165	0.10	0.03	0.37
(-)-Epicatechin	Flavonoids/Flavanols	Food	456	123	0.21	0.08	0.55
Resveratrol	Stilbenes	Food	429	52	0.09	0.02	0.54
Tyrosol	Tyrosols	Food	457	0	0.80	0.10	5.25
Hydroxytyrosol	Tyrosols	Food	474	0	2.44	0.75	12.85
Enterodiol	Lignans	Microbiota	433	22	0.37	0.09	1.73
Enterolactone	Lignans	Microbiota	469	3	3.12	0.54	12.22

LOQ, limit of quantification; P, percentile

^1^The main origin of the phenolic compound in urine is indicated. Food: the compound present in food is directly absorbed in the gut or it is absorbed after hydrolysis of the corresponding glycosides or esters. Microbiota: the compound results from the transformation by the microbiota of food polyphenols and/or eventually other food or endogenous compounds. Endogenous: the compounds results from the *O*-methylation of phenolic compounds of food or endogenous origin.

^2^Number of samples in which each phenolic compound was firmly identified.

**Table 3:** Urinary polyphenols most highly correlated to recent food intake in the EPIC cohort.

Food	Consumers (n)	Polyphenol (Spearman correlation coefficient)
Red wine	121	Gallic acid ethyl ester (0.69), resveratrol (0.59), gallic acid (0.48), hydroxytyrosol (0.43), tyrosol (0.36), (+)-catechin (0.34), *p*-coumaric acid (0.27), 4-hydroxyphenylacetic acid (0.19), 3,4-dihydroxyphenylacetic acid (0.15)
Coffee	410	Caffeic acid (0.65), protocatechuic acid (0.60), ferulic acid (0.58), *m*-coumaric acid (0.53), 3,4-dihydroxyphenylpropionic acid (0.51), 3-hydroxybenzoic acid (0.39), vanillic acid (0.31)
Tea	117	Gallic acid (0.38), (−)-epicatechin (0.30), (+)-catechin (0.22), quercetin (0.19)
Chocolate	111	(−)-Epicatechin (0.22), vanillic acid (0.15)
Citrus fruits	185	Hesperetin (0.60), naringenin (0.56), kaempferol (0.33)
Citrus juices	131	Hesperetin (0.15), naringenin (0.15), kaempferol (0.10)
Apple and pear	226	Phloretin (0.40), (−)-epicatechin (0.20), 3,4-dihydroxyphenylacetic acid (0.19), homovanillic acid (0.16)
Berries	42	*p*-Coumaric acid (0.20), (+)-catechin (0.19)
Onion, garlic	220	Quercetin (0.17), apigenin (0.11), isorhamnetin (0.10)
Olive oil	238	Hydroxytyrosol (0.36), tyrosol (0.31), 3,4-dihydroxyphenylacetic acid (0.17), apigenin (0.17)
Olives	44	Hydroxytyrosol (0.34), 3,4-dihydroxyphenylacetic acid (0.29), homovanillic acid (0.22), tyrosol (0.11)
Bread, non-white	260	3,5-Dihydroxybenzoic acid (0.45), 3,5-dihydroxyphenylpropionic acid (0.43), enterolactone (0.25), daidzein (0.20), enterodiol (0.20), genistein (0.19), m-coumaric acid (0.16), ferulic acid (0.13)
Breakfast cereals	32	3,5-Dihydroxybenzoic acid (0.17), 3,5-dihydroxyphenylpropionic acid (0.16), daidzein (0.15), equol (0.08), enterolactone (0.08)
Soya products	9	Genistein (0.17), daidzein (0.10)

The top two to nine polyphenols (out of 34 measured polyphenols) most highly correlated with the intake of each food group are listed. The number of reported correlations for each food group was based on current knowledge on polyphenol food composition and polyphenol metabolism. Some additional polyphenols may also be correlated to intake of each food, but they were excluded if not known as a component of the food considered or as a possible metabolite derived from a component of this food.
