# Supplementary material for: Urinary excretions of 34 dietary polyphenols and their associations with lifestyle factors in the EPIC cohort study
Source: Sci Rep. 2016 Jun 7;6:26905. doi: 10.1038/srep26905 (PMC4895229; doi:10.1038/srep26905)
Supplement: Supplementary Information [file srep26905-s1.pdf]

Supplementary material

Urinary excretions of 34 dietary polyphenols and their associations with lifestyle factors in the EPIC cohort study

Raul Zamora-Ros, David Achaintre, Joseph A. Rothwell, Sabina Rinaldi, Nada Assi, Pietro Ferrari, Michael Leitzmann, Marie-Christine Boutron-Ruault, Guy Fagherazzi, Aurélie Auffret, Tilman Kühn, Verena Katzke, Heiner Boeing, Antonia Trichopoulou, Androniki Naska, Effie Vasilopoulou, Domenico Palli, Sara Grioni, Amalia Mattiello, Rosario Tumino, Fulvio Ricceri, Nadia Slimani, Isabelle Romieu, Augustin Scalbert

Supplementary Table 1a. Urinary polyphenol concentrations by study centre in the EPIC cohort.

Urinary polyphenols (μmol/24h)	Florence (n=45)				Varese (n=51)				Ragusa (n=17)				Turin (n=42)				Naples (n=20)			
	N	Median	10th	90th	N	Median	10th	90th	N	Median	10th	90th	N	Median	10th	90th	N	Median	10th	90th
4-Hydroxybenzoic acid	44	18.0	9.56	28.8	51	17.9	9.60	31.0	17	21.8	14.4	36.5	42	21.1	12.8	57.4	20	18.2	9.56	30.6
3-Hydroxybenzoic acid	45	1.61	0.58	3.75	51	1.27	0.60	3.88	17	1.98	0.77	6.52	42	1.95	0.97	3.90	20	1.95	0.94	8.04
Protocatechuic acid	45	3.08	1.88	6.09	51	2.95	1.84	5.16	17	4.53	2.46	6.85	42	3.56	2.30	5.98	20	3.34	1.26	5.07
Gallic acid	31	0.84	0.37	2.38	41	0.96	0.25	1.97	14	0.67	0.36	2.06	31	0.84	0.36	2.54	15	0.95	0.23	2.25
Vanillic acid	45	35.8	14.3	63.2	46	34.0	17.6	140	17	43.0	17.7	124	38	47.3	18.3	117.7	20	33.0	15.1	74.9
3,5-Dihydroxybenzoic acid	45	2.61	1.66	6.22	49	2.80	1.39	4.52	17	6.67	2.93	12.4	42	2.76	1.40	7.90	20	3.81	1.42	15.6
Gallic acid ethyl ester	45	0.45	0.08	5.00	49	0.53	0.09	3.97	15	0.52	0.07	4.20	41	0.37	0.09	4.47	18	0.29	0.08	4.18
4-Hydroxyphenylacetic acid	44	143	95.2	232	51	198	108	405	17	179	112	325	42	167	103	352	20	134	83.6	296
3-Hydroxyphenylacetic acid	31	40.0	23.5	90.8	27	41.5	10.1	95.5	5	29.8	0.1	68.6	28	55.5	28.0	91.5	17	43.2	11.6	103
3,4-Dihydroxiphenylacetic acid	45	5.35	3.32	9.13	51	5.55	3.36	9.68	17	9.35	5.13	24.0	42	6.51	4.56	15.4	20	5.49	3.28	21.9
Homovanillic acid	45	26.0	16.5	41.1	51	24.7	18.0	37.0	17	28.5	18.5	51.0	42	26.8	19.9	40.1	20	24.6	15.2	60.6
3,4-Dihydroxyphenylpropionic acid	45	8.49	3.90	21.3	50	8.06	2.92	18.1	13	14.3	8.30	35.6	40	7.68	3.32	38.0	19	11.1	2.63	23.8
3,5-Dihydroxyphenylpropionic acid	45	8.45	4.52	19.6	51	7.30	3.46	13.3	17	18.5	6.17	29.5	42	8.33	3.65	15.6	20	11.0	3.53	23.5
p-Coumaric acid	45	1.83	0.75	3.36	51	1.96	0.92	3.96	17	3.33	1.45	6.27	42	2.83	1.39	6.10	19	1.69	0.78	4.47
m-Coumaric acid	44	1.58	0.55	5.07	50	1.64	0.47	4.72	17	2.54	0.70	8.93	39	2.42	0.67	7.37	20	1.67	0.39	4.43
Caffeic acid	45	3.87	1.98	7.70	51	3.97	1.98	7.59	17	6.85	3.15	15.9	42	5.02	3.12	9.14	20	5.76	1.85	7.38
Ferulic acid	45	40.4	19.2	64.4	51	42.3	19.7	68.5	17	59.2	16.2	91.4	40	40.1	16.8	70.7	20	42.5	19.8	63.6
Kaempferol	36	0.10	0.06	0.40	36	0.12	0.05	0.27	15	0.23	0.08	0.51	40	0.12	0.05	0.30	18	0.09	0.05	0.42
Quercetin	43	0.51	0.27	0.85	46	0.48	0.21	1.17	13	0.43	0.22	0.93	39	0.44	0.22	0.99	20	0.41	0.23	0.87
Isorhamnetin	44	0.49	0.27	1.07	50	0.46	0.24	1.04	16	0.53	0.19	1.29	40	0.48	0.28	0.98	20	0.56	0.25	1.57
Apigenin	40	0.13	0.04	0.53	51	0.06	0.02	0.31	16	0.15	0.02	1.16	40	0.07	0.01	0.30	17	0.07	0.01	0.16
Naringenin	44	2.24	0.57	18.7	50	2.61	0.79	12.8	17	9.93	1.54	18.0	42	1.42	0.29	7.80	20	1.78	0.53	5.52
Hesperetin	45	2.67	0.14	12.4	49	2.89	0.20	13.9	17	7.80	1.01	20.4	41	0.56	0.11	8.29	20	0.68	0.16	5.99
Daidzein	34	0.48	0.14	4.98	40	0.73	0.16	8.77	10	0.16	0.09	2.23	28	0.81	0.18	5.37	15	0.37	0.12	6.55

Genistein	38	0.11	0.04	0.92	41	0.18	0.07	1.04	9	0.10	0.06	0.51	38	0.10	0.03	0.65	19	0.12	0.01	0.64
Equol	37	0.05	0.01	0.12	46	0.06	0.01	0.15	15	0.06	0.01	0.10	35	0.05	0.01	0.14	16	0.07	0.04	0.16
Phloretin	45	0.30	0.15	0.87	51	0.35	0.17	0.97	17	0.62	0.14	1.14	42	0.39	0.19	1.50	20	0.34	0.16	0.88
(+)-Catechin	42	0.10	0.02	0.26	49	0.13	0.03	0.31	16	0.08	0.03	0.26	42	0.15	0.04	0.67	19	0.06	0.02	0.55
(-)-Epicatechin	42	0.18	0.05	0.34	48	0.21	0.09	0.46	14	0.19	0.08	0.36	40	0.25	0.10	0.70	20	0.17	0.06	0.48
Resveratrol	40	0.17	0.03	0.84	46	0.15	0.03	0.95	16	0.19	0.04	1.21	38	0.15	0.02	0.69	18	0.09	0.03	0.84
Tyrosol	45	1.60	0.57	6.00	49	1.78	0.21	5.50	17	2.56	0.26	7.31	39	1.42	0.32	5.88	19	0.67	0.16	2.16
Hydroxytyrosol	45	3.76	1.28	11.4	51	3.78	0.83	9.76	17	6.23	2.32	33.6	41	4.52	1.50	20.4	20	4.27	1.33	53.9
Enterodiol	40	0.36	0.07	0.69	45	0.21	0.05	1.04	16	0.25	0.06	0.63	38	0.32	0.05	1.42	19	0.27	0.06	1.35
Enterolactone	45	2.01	0.54	6.08	51	1.64	0.36	4.09	17	2.44	0.46	6.01	42	2.73	0.32	5.45	20	2.08	0.43	4.60

Supplementary Table 1b. Urinary polyphenol concentrations by study centre in the EPIC cohort.

Urinary polyphenols (µmol/24h)	Greece (n=56)				Ile-de-France (n=67)				Heidelberg (n=59)				Potsdam (n=118)				Fold change	P-value
	N	Median	10th	90th	N	Median	10th	90th	N	Median	10th	90th	N	Median	10th	90th		
4-Hydroxybenzoic acid	55	11	5.1	26	67	19	11.6	36	59	20	13.3	33	118	23	12.3	44	2.0	<0.001
3-Hydroxybenzoic acid	56	0.9	0.2	3.2	67	2.3	0.9	5.5	59	3.5	0.8	10	118	2.8	1.1	9.0	3.7	0.006
Protocatechuic acid	56	1.92	0.89	3.06	67	3.22	2.03	5.65	59	4.90	2.47	8.86	118	4.42	2.34	6.86	2.6	<0.001
Gallic acid	44	0.3	0.1	0.6	49	0.8	0.4	2.8	37	0.8	0.4	2.2	74	0.8	0.3	2.4	3.3	<0.001
Vanillic acid	56	17.4	5.1	61.3	67	26.7	13.9	63.7	59	39.2	22.0	104	116	48.6	24.8	97.3	2.8	<0.001
3,5-Dihydroxybenzoic acid	56	2.4	0.9	6.1	66	3.1	1.4	8.6	59	7.8	3.1	16.2	114	7.5	3.3	13.5	3.2	<0.001
Gallic acid ethyl ester	53	0.1	0.09	0.3	65	0.5	0.1	2.5	58	0.2	0.1	1.2	106	0.1	0.1	1.1	4.1	<0.001
4-Hydroxyphenylacetic acid	56	109.37	64.08	221.1	67	155.06	90.54	247.1	59	182.2	93.14	363.6	118	171.0	98.50	357.9	1.8	<0.001
3-Hydroxyphenylacetic acid	30	30.76	14.13	90.7	55	59.10	33.78	95.0	33	45.3	19.82	131.5	73	43.6	23.61	91.9	2.0	<0.001
3,4-Dihydroxiphenylacetic acid	56	3.98	2.15	12.6	67	3.82	2.50	8.83	59	6.38	3.36	12.8	118	4.85	2.97	7.36	2.4	<0.001
Homovanillic acid	56	16.93	10.70	40.93	67	21.95	16.03	41.18	59	27.42	14.38	40.8	118	23.88	16.51	33.5	1.7	<0.001
3,4-Dihydroxyphenylpropionic acid	56	5.70	1.52	21.60	66	6.74	3.50	17.77	56	16.63	5.77	47.8	108	11.42	3.43	32.4	2.9	<0.001
3,5-Dihydroxyphenylpropionic acid	56	8.08	3.85	15.0	67	8.18	4.00	21.61	59	19.85	9.27	40.3	116	18.47	9.48	34.0	2.7	<0.001
p-Coumaric acid	56	1.25	0.51	4.1	67	2.22	1.32	4.68	57	2.64	1.33	6.1	110	2.27	1.21	4.35	2.7	<0.001
m-Coumaric acid	54	1.04	0.11	2.40	67	2.04	0.50	5.73	59	4.51	1.71	19.1	117	4.36	1.13	17.6	4.4	<0.001
Caffeic acid	56	2.76	1.08	5.40	67	3.72	2.30	6.96	59	7.59	2.66	14.99	118	6.17	2.36	11.98	2.7	<0.001
Ferulic acid	56	24.86	8.92	45.05	67	33.19	15.86	63.10	58	53.93	29.26	94.4	116	54.78	29.45	96.79	2.4	<0.001
Kaempferol	50	0.11	0.04	0.25	64	0.12	0.04	0.3	52	0.13	0.05	0.24	97	0.13	0.06	0.27	2.5	<0.001
Quercetin	55	0.40	0.20	0.84	67	0.53	0.25	1.17	53	0.59	0.28	1.1	108	0.56	0.24	1.3	1.5	<0.001
Isorhamnetin	56	0.52	0.29	1.04	66	0.50	0.36	1.69	58	0.56	0.30	1.28	112	0.53	0.25	1.02	1.2	<0.001
Apigenin	53	0.09	0.02	0.36	66	0.04	0.01	0.24	55	0.08	0.01	0.42	110	0.06	0.01	0.24	4.0	<0.001
Naringenin	55	1.54	0.43	8.60	67	0.91	0.33	10.84	59	1.55	0.52	8.15	116	1.81	0.50	7.13	10.9	<0.001
Hesperetin	55	0.66	0.16	7.59	66	0.46	0.13	1.46	59	0.91	0.16	4.50	117	1.34	0.16	6.98	17.0	0.47
Daidzein	47	0.18	0.04	3.17	61	1.04	0.20	6.68	56	2.38	0.71	17.52	116	2.35	0.44	10.97	15.3	0.018

Genistein	42	0.08	0.02	0.38	57	0.21	0.08	1.05	58	0.36	0.11	2.60	111	0.37	0.11	1.57	4.5	<0.001
Equol	40	0.01	0.01	0.10	57	0.04	0.01	0.14	48	0.06	0.01	0.41	103	0.05	0.01	0.12	5.1	<0.001
Phloretin	56	0.27	0.17	0.74	67	0.31	0.15	0.91	59	0.41	0.18	1.37	118	0.52	0.19	1.61	2.3	<0.001
(+)-Catechin	55	0.04	0.03	0.12	65	0.14	0.04	0.41	52	0.15	0.04	0.48	112	0.10	0.03	0.36	3.4	<0.001
(-)-Epicatechin	55	0.10	0.07	0.27	65	0.28	0.09	0.61	58	0.29	0.08	0.88	114	0.26	0.10	0.59	2.8	<0.001
Resveratrol	50	0.04	0.02	0.10	64	0.14	0.03	0.48	53	0.09	0.04	0.24	104	0.08	0.03	0.54	5.4	0.09
Tyrosol	56	0.88	0.11	4.43	66	0.66	0.11	2.77	54	0.52	0.08	6.14	112	0.35	0.07	4.40	7.4	<0.001
Hydroxytyrosol	56	2.13	0.75	23.00	67	1.60	0.68	6.98	59	2.56	0.71	16.11	118	1.40	0.50	4.19	4.4	<0.001
Enterodiol	49	0.39	0.06	4.12	61	0.40	0.15	1.18	58	0.59	0.13	4.35	107	0.44	0.13	1.73	2.8	<0.001
Enterolactone	52	2.43	0.35	14.49	67	3.76	1.07	10.82	58	5.04	1.06	23.02	117	5.11	0.82	17.93	3.1	<0.001

Significant P value <0.001 (0.05/35) using Kruskal-Wallis test

Supplementary Table 2. Urinary polyphenol concentrations by sex in the EPIC cohort

Urinary polyphenols (μmol/24h)	Men				Women				Fold change	P-value
	N	Median	10th	90th	N	Median	10th	90th		
4-Hydroxybenzoic acid	197	21.6	16.0	26.9	276	18.0	12.9	25.0	1.2	<0.001
3-Hydroxybenzoic acid	198	2.14	1.09	3.90	277	1.94	1.13	3.49	1.1	0.31
Protocatechuic acid	198	3.85	2.63	5.44	277	3.21	2.49	4.57	1.2	0.002
Gallic acid	152	0.83	0.37	1.61	184	0.65	0.40	1.07	1.3	0.16
Vanillic acid	190	43.3	29.1	74.5	274	30.6	20.4	50.2	1.4	<0.001
3,5-Dihydroxybenzoic acid	193	4.17	2.64	7.97	275	3.95	2.13	6.83	1.1	0.028
Gallic acid ethyl ester	191	0.19	0.12	1.20	259	0.18	0.11	0.66	1.1	0.11
4-Hydroxyphenylacetic acid	197	191	140	268	277	135	108	188	1.4	<0.001
3-Hydroxyphenylacetic acid	96	43.1	27.7	65.9	203	50.0	31.1	69.1	1.2	0.08
3,4-Dihydroxiphenylacetic acid	198	5.79	4.25	7.84	277	4.75	3.45	6.70	1.2	<0.001
Homovanillic acid	198	26.9	21.6	32.3	277	22.1	18.1	28.5	1.2	<0.001
3,4-Dihydroxyphenylpropionic acid	187	11.1	5.21	19.1	266	8.43	5.03	14.9	1.3	0.039
3,5-Dihydroxyphenylpropionic acid	197	13.3	7.69	22.2	276	10.3	6.08	17.6	1.3	0.001
p-Coumaric acid	190	2.44	1.60	3.73	274	1.96	1.33	2.93	1.2	<0.001
m-Coumaric acid	194	2.70	1.20	6.14	273	2.05	1.06	4.31	1.3	0.003
Caffeic acid	198	5.33	3.15	8.48	277	4.43	3.06	6.75	1.2	0.032
Ferulic acid	195	51.0	33.4	72.5	275	36.9	25.0	51.0	1.4	<0.001
Kaempferol	162	0.13	0.09	0.20	246	0.12	0.07	0.19	1.1	0.06
Quercetin	184	0.55	0.40	0.85	260	0.47	0.32	0.68	1.2	<0.001
Isorhamnetin	190	0.58	0.39	0.85	272	0.49	0.36	0.74	1.2	0.037
Apigenin	184	0.07	0.03	0.16	264	0.08	0.02	0.16	1.0	0.72
Naringenin	195	2.23	0.92	5.59	275	1.44	0.73	3.31	1.6	<0.001
Hesperetin	195	1.60	0.44	5.47	274	0.81	0.22	2.74	2.0	<0.001
Daidzein	173	1.85	0.39	3.91	234	1.00	0.39	2.61	1.9	0.06
Genistein	169	0.29	0.11	0.69	244	0.18	0.09	0.37	1.6	0.003
Equol	164	0.05	0.02	0.08	233	0.05	0.02	0.09	1.0	0.68
Phloretin	198	0.47	0.25	0.82	277	0.34	0.23	0.66	1.4	0.002
(-)-Epicatechin	192	0.23	0.14	0.38	264	0.19	0.10	0.32	1.2	0.003
(+)-Catechin	187	0.12	0.06	0.23	265	0.08	0.04	0.18	1.5	0.002
Resveratrol	179	0.10	0.05	0.33	250	0.09	0.04	0.19	1.2	0.010
Tyrosol	185	1.58	0.36	3.92	272	0.65	0.25	1.43	2.4	<0.001
Hydroxytyrosol	197	2.82	1.37	6.81	277	2.21	1.10	4.52	1.3	0.043
Enterodiol	181	0.34	0.18	0.69	252	0.41	0.21	0.79	1.2	0.10
Enterolactone	195	3.12	1.20	6.54	274	3.13	1.65	6.08	1.0	0.69

Significant P value <0.001 (0.05/35) using Kruskal-Wallis test

Supplementary Table 3. Urinary polyphenol concentrations by the highest educational level in the EPIC cohort

Urinary polyphenols (μmol/24h)	None and primary school				Technical and secondary school				University				Fold change	P-value
	N	Median	10th	90th	N	Median	10th	90th	N	Median	10th	90th		
4-Hydroxybenzoic acid	181	17.4	12.1	23.6	170	20.9	15.8	27.6	122	19.9	15.0	27.1	1.2	<0.001
3-Hydroxybenzoic acid	182	1.84	0.98	3.21	171	2.24	1.21	3.90	122	2.30	1.28	4.37	1.3	0.004
Protocatechuic acid	182	3.06	2.03	4.65	171	3.56	2.88	4.90	122	3.83	2.58	5.20	1.3	0.002
Gallic acid	128	0.56	0.32	1.10	121	0.67	0.41	1.15	87	0.90	0.54	1.92	1.6	<0.001
Vanillic acid	181	32.6	20.3	61.3	166	36.4	24.8	57.7	117	40.0	25.2	63.5	1.2	0.26
3,5-Dihydroxybenzoic acid	181	3.57	2.13	6.22	168	4.06	2.54	7.12	119	4.79	2.63	8.92	1.3	0.003
Gallic acid ethyl ester	171	0.18	0.12	0.87	161	0.21	0.11	0.60	118	0.19	0.11	1.07	1.1	0.81
4-Hydroxyphenylacetic acid	182	142	108	200	170	171	117	239	122	156	116	233	1.2	0.022
3-Hydroxyphenylacetic acid	111	41.8	27.1	62.8	104	50.5	33.6	68.9	84	47.2	31.4	79.0	1.2	0.09
3,4-Dihydroxiphenylacetic acid	182	4.90	3.62	7.55	171	5.54	3.83	7.41	122	5.01	3.81	6.69	1.1	0.32
Homovanillic acid	182	23.3	17.6	30.6	171	25.2	19.8	31.9	122	23.6	20.3	28.8	1.1	0.16
3,4-Dihydroxyphenylpropionic acid	178	9.05	4.97	17.3	160	9.41	5.58	14.2	115	9.76	4.67	18.1	1.1	0.99
3,5-Dihydroxyphenylpropionic acid	182	9.80	6.14	16.6	170	11.4	7.12	19.3	121	14.2	7.89	24.6	1.4	0.001
p-Coumaric acid	181	1.80	1.17	3.17	168	2.10	1.49	3.07	115	2.33	1.69	3.44	1.3	0.007
m-Coumaric acid	177	1.83	1.00	3.92	169	2.64	1.35	5.44	121	2.49	1.11	5.07	1.4	0.005
Ferulic acid	181	38.7	25.0	56.0	169	44.4	30.7	58.4	120	47.0	29.4	70.6	1.2	0.002
Caffeic acid	182	4.23	2.62	6.90	171	5.25	3.34	7.50	122	4.77	3.19	8.43	1.2	0.06
Kaempferol	156	0.12	0.08	0.21	144	0.12	0.08	0.18	108	0.12	0.08	0.21	1.0	0.71
Quercetin	171	0.49	0.33	0.74	155	0.54	0.36	0.78	118	0.52	0.37	0.76	1.1	0.59
Isorhamnetin	180	0.53	0.37	0.80	167	0.52	0.38	0.75	115	0.51	0.36	0.85	1.1	0.82
Apigenin	174	0.09	0.04	0.22	159	0.06	0.02	0.13	115	0.07	0.02	0.15	1.4	0.009
Naringenin	180	1.98	0.88	5.10	168	1.60	0.79	4.67	122	1.38	0.77	3.40	1.4	0.11
Hesperetin	180	1.28	0.25	4.33	168	1.05	0.33	3.50	121	0.77	0.22	2.86	1.7	0.09
Daidzein	149	0.60	0.18	1.99	147	1.83	0.59	4.20	111	1.81	0.60	3.93	3.1	<0.001

Genistein	155	0.17	0.07	0.35	149	0.23	0.11	0.53	109	0.30	0.11	0.61	1.8	0.005
Equol	153	0.04	0.02	0.08	142	0.05	0.03	0.09	102	0.05	0.03	0.10	1.2	0.12
Phloretin	182	0.36	0.24	0.72	171	0.37	0.23	0.68	122	0.39	0.25	0.74	1.1	0.79
(+)-Catechin	171	0.08	0.04	0.17	164	0.12	0.05	0.21	117	0.11	0.05	0.23	1.5	0.045
(-)-Epicatechin	174	0.18	0.10	0.33	162	0.22	0.14	0.36	120	0.25	0.13	0.41	1.4	0.018
Resveratrol	162	0.08	0.04	0.21	154	0.09	0.05	0.21	113	0.12	0.06	0.26	1.6	0.07
Tyrosol	176	0.76	0.26	1.88	167	0.77	0.22	2.43	114	0.86	0.34	2.27	1.1	0.39
Hydroxytyrosol	182	2.46	1.20	5.43	170	2.25	1.10	5.61	122	2.44	1.37	4.19	1.1	0.77
Enterodiol	166	0.36	0.17	0.82	156	0.36	0.16	0.70	111	0.40	0.26	0.72	1.1	0.26
Enterolactone	179	2.45	1.23	4.58	169	3.15	1.30	6.01	121	4.50	2.05	8.57	1.8	<0.001

Significant P value <0.001 (0.05/35) using Kruskal-Wallis test

Supplementary Table 4. Urinary polyphenol concentrations by total energy intake in the EPIC cohort

Urinary polyphenols (µmol/24h)	<1780kcal/d				1780-2375kcal/d				>2375kcal/d				Fold change	P-value
	N	Median	10th	90th	N	Median	10th	90th	N	Median	10th	90th		
4-Hydroxybenzoic acid	157	17.4	12.5	24.2	158	20.0	13.9	26.0	158	20.4	15.9	26.7	1.2	0.013
3-Hydroxybenzoic acid	158	1.85	1.09	3.62	159	1.98	1.07	3.53	158	2.30	1.23	3.97	1.2	0.12
Protocatechuic acid	158	3.05	2.17	4.12	159	3.47	2.41	5.05	158	3.96	2.87	5.68	1.3	<0.001
Gallic acid	108	0.51	0.33	0.97	111	0.72	0.42	1.39	117	0.84	0.51	1.56	1.7	0.023
Vanillic acid	158	29.5	18.3	49.1	156	32.7	22.6	55.7	150	43.3	28.5	73.2	1.5	<0.001
3,5-Dihydroxybenzoic acid	157	3.82	2.12	6.27	155	4.04	2.39	7.80	156	4.19	2.67	8.30	1.1	0.023
Gallic acid ethyl ester	147	0.16	0.11	0.42	150	0.21	0.10	1.02	153	0.23	0.12	1.16	1.4	0.046
4-Hydroxyphenylacetic acid	158	134	103	192	158	153	111	210	158	187	136	261	1.4	<0.001
3-Hydroxyphenylacetic acid	111	44.2	29.6	66.7	103	44.6	29.2	65.8	85	53.3	32.4	70.7	1.2	0.28
3,4-Dihydroxiphenylacetic acid	158	4.82	3.41	6.13	159	5.00	3.73	7.35	158	5.59	4.14	8.09	1.2	0.001
Homovanillic acid	158	21.8	17.9	27.5	159	25.1	20.0	31.5	158	25.8	20.7	32.1	1.2	<0.001
3,4-Dihydroxyphenylpropionic acid	150	8.24	4.86	14.1	155	9.05	5.02	17.3	148	10.7	5.70	18.1	1.3	0.11
3,5-Dihydroxyphenylpropionic acid	156	10.2	5.82	17.3	159	12.5	7.12	20.8	158	11.7	7.69	20.3	1.2	0.07
p-Coumaric acid	155	1.95	1.25	2.90	154	2.03	1.33	3.14	155	2.38	1.68	3.88	1.2	<0.001
m-Coumaric acid	158	2.03	0.94	4.35	153	2.24	1.13	4.70	156	2.63	1.45	6.19	1.3	0.010
Caffeic acid	158	4.17	3.08	6.90	159	4.64	2.81	6.96	158	5.41	3.29	8.41	1.3	0.032
Ferulic acid	156	37.8	24.7	50.7	158	40.7	29.0	59.4	156	49.1	29.6	66.3	1.3	<0.001
Kaempferol	133	0.12	0.07	0.18	140	0.12	0.08	0.20	135	0.13	0.09	0.21	1.1	0.26
Quercetin	144	0.45	0.31	0.69	153	0.52	0.37	0.75	147	0.55	0.38	0.81	1.2	0.039
Isorhamnetin	155	0.51	0.39	0.77	156	0.54	0.35	0.81	151	0.53	0.39	0.78	1.1	0.84
Apigenin	150	0.07	0.03	0.14	150	0.07	0.02	0.16	148	0.08	0.02	0.18	1.2	0.77
Naringenin	157	1.47	0.75	4.80	158	1.62	0.92	3.35	155	1.94	0.79	4.57	1.3	0.71
Hesperetin	155	0.99	0.24	4.03	158	0.99	0.32	3.36	156	1.08	0.23	3.44	1.1	0.93
Daidzein	133	0.81	0.22	2.26	134	1.89	0.41	3.93	140	1.64	0.45	3.61	2.3	0.006
Genistein	129	0.17	0.08	0.35	145	0.23	0.10	0.60	139	0.28	0.11	0.59	1.6	0.008

Equol	129	0.04	0.02	0.06	132	0.05	0.03	0.09	136	0.06	0.03	0.10	1.7	<0.001
Phloretin	158	0.36	0.24	0.69	159	0.38	0.22	0.80	158	0.36	0.25	0.70	1.1	0.75
(+)-Catechin	155	0.08	0.04	0.18	148	0.10	0.04	0.21	149	0.13	0.06	0.22	1.5	0.006
(-)-Epicatechin	153	0.18	0.10	0.31	149	0.20	0.13	0.36	154	0.23	0.13	0.37	1.3	0.018
Resveratrol	141	0.08	0.03	0.19	140	0.08	0.05	0.20	148	0.11	0.06	0.31	1.4	0.003
Tyrosol	153	0.64	0.21	1.68	154	0.77	0.25	1.89	150	1.10	0.40	3.44	1.7	0.005
Hydroxytyrosol	158	2.08	1.08	3.48	159	2.69	1.19	5.22	157	2.99	1.43	6.98	1.4	0.006
Enterodiol	140	0.37	0.18	0.78	148	0.36	0.17	0.69	145	0.39	0.22	0.72	1.1	0.73
Enterolactone	154	3.01	1.34	6.04	158	3.09	1.50	5.65	157	3.24	1.56	7.11	1.1	0.62

Significant P value <0.001 (0.05/35) using Kruskal-Wallis test

Supplementary Table 5. Urinary polyphenol concentrations by baseline alcohol intake in the EPIC cohort

Urinary polyphenols (µmol/24h)	<0.1g/d				0.1-20g/d				>20g/d				Fold change	P-value
	N	Median	10th	90th	N	Median	10th	90th	N	Median	10th	90th		
4-Hydroxybenzoic acid	151	17.6	12.1	23.9	189	19.5	14.1	25.3	133	21.4	15.8	27.8	1.2	0.009
3-Hydroxybenzoic acid	151	1.57	0.84	3.48	190	2.11	1.21	3.59	134	2.36	1.34	4.60	1.5	0.001
Protocatechuic acid	151	2.90	1.99	4.50	190	3.55	2.63	5.05	134	3.83	2.90	5.61	1.3	<0.001
Gallic acid	91	0.48	0.29	0.72	133	0.68	0.38	1.11	112	1.08	0.64	1.98	2.3	<0.001
Vanillic acid	149	31.9	19.0	56.8	187	35.5	22.8	57.7	128	42.1	27.2	64.4	1.3	0.018
3,5-Dihydroxybenzoic acid	149	4.09	2.13	7.72	186	4.48	2.61	7.95	133	3.65	2.38	6.75	1.2	0.262
Gallic acid ethyl ester	137	0.13	0.09	0.17	182	0.19	0.11	0.65	131	0.92	0.25	2.83	7.2	<0.001
4-Hydroxyphenylacetic acid	151	121	92	171	190	151	115	206	133	204	158	273	1.7	<0.001
3-Hydroxyphenylacetic acid	101	48.4	28.1	66.1	126	44.3	29.5	66.5	72	52.8	33.1	69.7	1.2	0.34
3,4-Dihydroxiphenylacetic acid	151	4.58	3.16	6.26	190	4.91	3.67	6.83	134	6.24	4.86	8.10	1.4	<0.001
Homovanillic acid	151	22.9	17.3	28.9	190	22.9	19.0	29.8	134	26.4	21.3	32.3	1.2	<0.001
3,4-Dihydroxyphenylpropionic acid	146	7.81	4.43	15.9	181	10.3	5.78	16.6	126	9.41	5.31	18.5	1.3	0.09
3,5-Dihydroxyphenylpropionic acid	150	10.7	6.14	19.6	189	12.6	7.59	19.8	134	10.5	6.88	18.5	1.2	0.34
p-Coumaric acid	150	1.63	1.01	2.41	184	2.19	1.40	3.04	130	2.82	1.95	4.72	1.7	<0.001
m-Coumaric acid	147	1.66	0.73	4.31	189	2.28	1.37	4.10	131	3.08	1.48	6.95	1.9	<0.001
Ferulic acid	151	36.9	24.6	51.8	187	42.5	29.7	61.6	132	45.6	30.4	61.8	1.2	<0.001
Caffeic acid	151	3.87	2.58	6.18	190	5.24	3.16	7.25	134	5.39	3.62	8.35	1.4	<0.001
Kaempferol	134	0.12	0.07	0.18	160	0.13	0.08	0.21	114	0.12	0.09	0.21	1.1	0.44
Quercetin	144	0.43	0.29	0.68	176	0.52	0.38	0.77	124	0.54	0.42	0.79	1.3	0.004
Isorhamnetin	149	0.52	0.36	0.74	182	0.52	0.36	0.79	131	0.53	0.39	0.85	1.0	0.72
Apigenin	140	0.08	0.02	0.18	180	0.07	0.03	0.16	128	0.08	0.02	0.15	1.1	0.88
Naringenin	149	1.46	0.75	3.25	190	1.90	0.87	4.66	131	1.60	0.82	4.58	1.3	0.30
Hesperetin	147	0.76	0.22	2.29	189	1.25	0.26	3.90	133	1.00	0.35	4.95	1.6	0.026
Daidzein	128	1.01	0.33	2.54	171	1.30	0.43	4.08	108	1.37	0.43	3.69	1.4	0.14

Genistein	128	0.18	0.08	0.43	172	0.26	0.12	0.63	113	0.21	0.10	0.38	1.5	0.050
Equol	124	0.05	0.02	0.08	167	0.05	0.02	0.09	106	0.05	0.02	0.08	1.1	0.75
Phloretin	151	0.34	0.22	0.72	190	0.39	0.25	0.79	134	0.36	0.25	0.65	1.1	0.41
(+)-Catechin	144	0.06	0.04	0.12	179	0.09	0.04	0.17	129	0.18	0.11	0.34	3.1	<0.001
(-)-Epicatechin	143	0.18	0.10	0.32	184	0.21	0.12	0.35	129	0.26	0.15	0.40	1.5	0.006
Resveratrol	134	0.05	0.03	0.08	174	0.10	0.05	0.19	121	0.25	0.10	0.54	4.8	<0.001
Tyrosol	151	0.36	0.13	0.98	189	0.60	0.27	1.57	117	3.25	1.69	5.88	9.0	<0.001
Hydroxytyrosol	151	1.48	0.84	2.68	190	2.05	1.16	3.89	133	6.07	3.19	11.5	4.1	<0.001
Enterodiol	139	0.31	0.17	0.72	172	0.46	0.21	0.78	122	0.35	0.19	0.69	1.5	0.28
Enterolactone	148	2.62	1.11	4.76	189	3.28	1.64	6.96	132	3.36	1.67	6.16	1.3	0.032
